# Professor Siliman

**Published:** 1853-09

**Authors:** 


### PROFESSOR SILLIMAN.

Professor Silliman, Sen., having resigned his professorship of Chemistry, Geology and Mineralogy in Yale College, is succeeded by his son, and his son-in-law, J. D. Dana, the former in the chair of Chemistry, and the latter in that of Geology and Mineralogy. This announcement, which has been extensively made, has created the impression that Prof. Silliman, Jr., has resigned the chair of Chemistry in the Universtty of Louisville. Such is not the fact. Prof. Silliman will deliver the Chemical Course in this institution, as usual, during the approaching winter, and will be in Louisville in time to give some lectures in the preliminary Course, which is to commence in October. No change in the organization of the Faculty has taken place during the present year, and we are happy to say, none seems likely.

